# Vitamin Nature: How Coronavirus Disease 2019 Has Highlighted Factors Contributing to the Frequency of Nature Visits in Flanders, Belgium

**DOI:** 10.3389/fpubh.2021.646568

**Published:** 2021-05-11

**Authors:** Aline Lenaerts, Sofie Heyman, Annelies De Decker, Laura Lauwers, Ann Sterckx, Roy Remmen, Hilde Bastiaens, Hans Keune

**Affiliations:** ^1^Chair Care and the Natural Living Environment, Centre for General Practice, Faculty of Medicine and Health Sciences, University of Antwerp, Antwerp, Belgium; ^2^Provincial Institute for Hygiene (PIH), Knowledge Centre for Environment and Health, Province of Antwerp, Antwerp, Belgium

**Keywords:** COVID-19, confinement measures, green space, nature, ecosystem service, citizen perceptions

## Abstract

Visiting nature is positively associated with physical and mental well-being. The role of nature became more pronounced during the coronavirus outbreak in the spring of 2020. Countries all over the world implemented confinement measures to reduce the transmission of the virus. These included but were not limited to the cancelation of public events, schools, and non-essential businesses and the prohibition of non-essential travels. However, going outside to exercise was recommended by the Belgian government. During this period, we conducted an online survey to determine if people visit nature more frequently than before and to identify the factors that contribute to this. The results are based on data from 11,352 participants in Flanders, Belgium. With the use of a bivariate and multiple regression analysis, results indicate that people visit nature more frequently than before and that nature helped to maintain social relationships during the coronavirus period. Gardens were reported to be the most popular place, followed by parks. More than half of the people experienced nature in a more positive way, and the belief that nature visits are important for general health increased. In addition, we found a positive association between nature visits and home satisfaction, as well as a positive association with subjective mental and physical health. Lastly, we identified several demographic factors contributing to the frequency of nature visits such as age, gender, and socioeconomic status. Our findings indicate the importance of nature visits for general well-being and highlight the need for nearby green infrastructure.

## Introduction

It is widely acknowledged that nature affects human health (1–3). Increasing empirical evidence demonstrates a positive relationship between nature and well-being (4–6), such as improved relaxation and restoration, enhanced immune function, improved air quality, social connectedness, and increased physical activity (7). These findings have led to more health-care research exploring the value of nature's contributions to (primary) health care (8, 9). The importance of contact with nature for human health became clear during the coronavirus pandemic (10). Previous research highlighted that contact with nature may be an effective strategy to cope with stress (11, 12) and emotion regulation (13). During the pandemic, a trend of people visiting nature more often could be observed in western countries (14–17).

There are different views regarding the interpretation of contact with nature in the literature. Frumkin et al. (7) argue that there are different ways of contact with nature: “varying by spatial scale, proximity, the sensory pathway through which nature is experienced (visual, auditory, etc.), the individual's activities and level of awareness while in a natural setting, and other factors” [as cited in Frumkin et al. (7)]. First of all, Frumkin and Fox (18) refer to contact with nature in buildings. These are plants, photos, or videos of natural environments as well as looking out on nature. This type of contact with nature is indirect (19). However, research shows that this type of contact with nature is also associated with improved health and well-being (7, 18, 20). Secondly, neighborhoods with a green environment such as trees and plants are also subject to contact with nature (18). This can be classified under incidental contact (19). A third and last type of contact with nature is the conscious search for a green environment such as a park, garden, forest, or nature reserve (18). The latter is described as intentional contact with nature (19).

This study focused on the intentional seeking of nature, and thus, we refer to “nature visit” instead of contact with nature. Nature was broadly defined: ranging from a green terrace/balcony or garden to nature in the environment such as a (city) park, nature reserve, forest, field, meadow, pond, river, sea, and beach. This definition was opted based on previous research that showed how benefits can differ based on the type of nature (21, 22).

### The Coronavirus Disease 2019 Pandemic: Confinement Measures in Belgium

The coronavirus was first reported in Wuhan, China, on December 31, 2019 (23, 24). On March 11, 2020, the WHO characterized the coronavirus disease as a pandemic (25, 26). To reduce the transmission of the virus, governments worldwide imposed exceptional confinement measures (27–29), which affected our daily life (30) and psychological health (31, 32). These regulations had a negative effect on people's social participation, life satisfaction, and sedentary behavior (33, 34).

Furthermore, changes in eating pattern were observed (35), including, among others, an increased consumption of unhealthy foods (34).

In Belgium, the measures implemented to deal with the first coronavirus outbreak took effect on March 14, 2020. These included, but were not limited to, keeping physical distance from other people; the prohibition of all recreational activities and public gatherings; closing of non-essential stores, bars, and restaurants; and mandatory working from home, where applicable. Going outdoors was only allowed for essential reasons. However, outdoor exercise was allowed and even recommended by the government. No restrictions were imposed in terms of distance from home. These regulations were gradually phased out starting May 3 (36).

With a growing body of evidence of proven health benefits from visiting nature (6, 8, 37), the current study aimed to investigate to which extent people visit nature (more often) when the first confinement measures where applicable in Flanders, Belgium, and how this contributes to their perceived general well-being and their perception of nature and health.

In sum, the key research questions of this study were as follows: (1) Do people visit nature more often than before the coronavirus disease 2019 (COVID-19) confinement measures? (2) Which factors influence the frequency of nature visits during the COVID-19 confinement measures in Flanders, Belgium? Due to the exceptional situation of the coronavirus, we started this research with an open mind. We focused on the presented research questions and the hypothesis that there would be a noticeable increase in nature visits due to the COVID-19 confinement measures.

To ensure clarity and consistency in this paper, we will refer to C19CM (COVID-19 confinement measures) to indicate the period in which confinement measures were imposed by the Belgian Government and the online survey was conducted. More specifically, this concerns the period between April 9 and 19.

## Materials and Methods

### Study Design and Sample

Data were gathered through a cross-sectional survey design. An online survey was launched using Socratos Survey Software. This study was conducted by the Chair of Care and Natural Living Environment of the Faculty of Medicine and Health Sciences of the University of Antwerp. The chair is funded by the Province of Antwerp. The Department of Environment, subdivision Sustainable Environmental and Nature Policy, and the PIH of the Province of Antwerp also contributed to the realization of this study. Participants were recruited by convenience sampling (38). The authors distributed the online survey in the network of the Chair, the University of Antwerp, and the Province through press announcements, email, and social media. Respondents were asked to complete the survey and subsequently distribute the survey further into their own network. Additionally, in the days and weeks after the initial launch, the survey call was communicated by Flemish newspapers and several radio and television networks.

**Figure 1:**
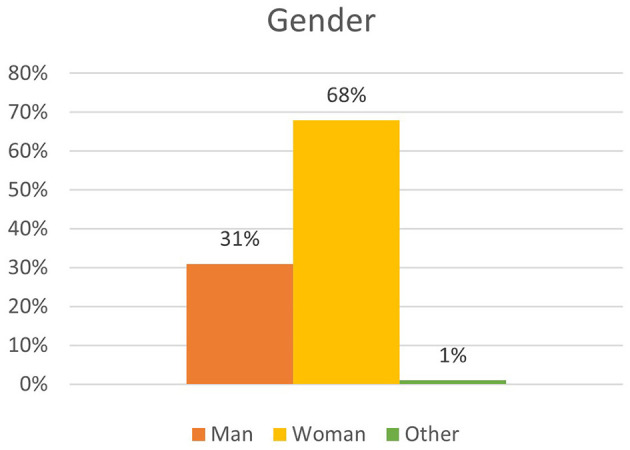
Overview of distribution by gender.

**Figure 2:**
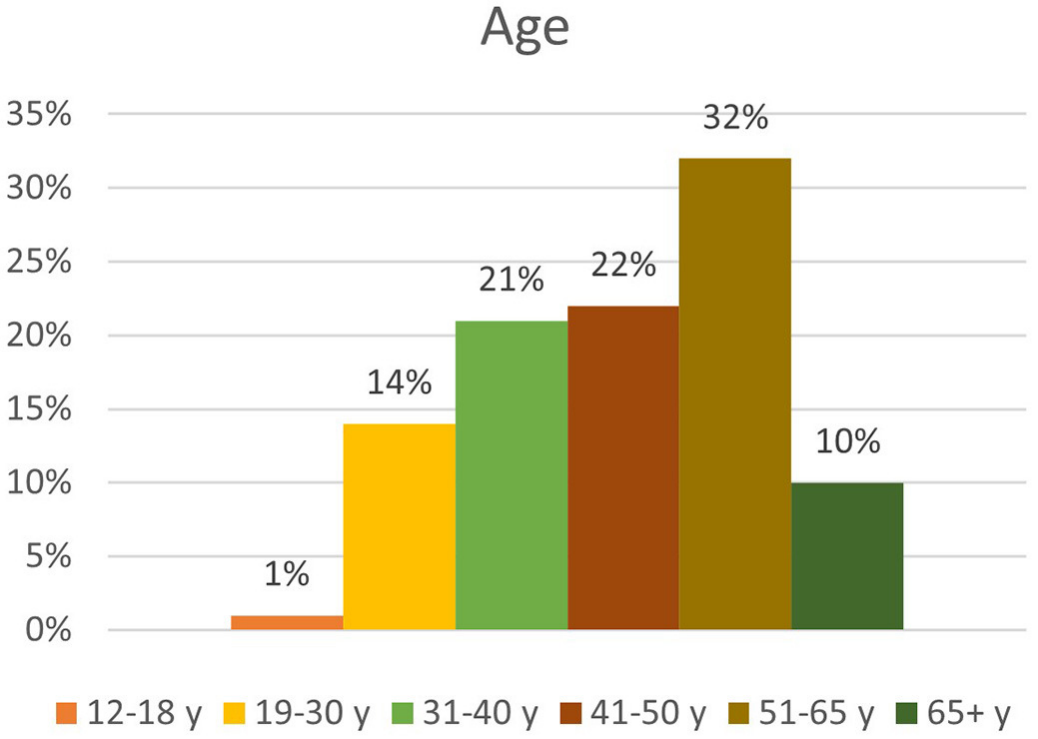
Overview of distribution by age.

**Figure 3:**
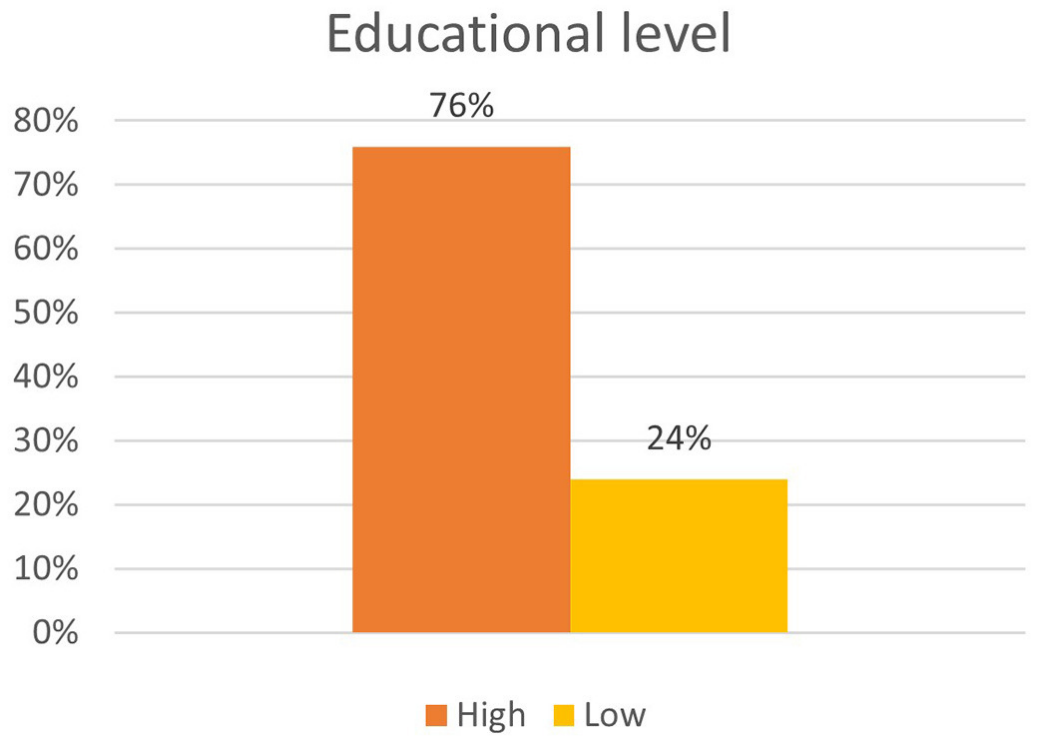
Overview of distribution by educational level.

A total of 11,352 participants completed the survey. The sample is not representative for the Flemish population, as there is a significant overrepresentation of female respondents, highly educated people, respondents living in the Province of Antwerp, and respondents reporting feeling healthy. An overview of the demographic factors can be found in Figure 1 (gender), Figure 2 (age) and Figure 3 (educational level).

### Ethics

Ethical approval was obtained from the Committee for Medical Ethics (CME) of Antwerp University Hospital (study 20/15/182/B3002020000062) on April 6, 2020. Prior to the start of the survey, participants had to confirm their informed consent. No incentives were promised for completing the survey. Participation was voluntary, and all respondents had the right to leave the survey at any point.

### Measurements

A pretest was conducted by 15 people. After their feedback was received, a few questions and response options were adjusted to precise questions and formulation in order to avoid misunderstandings. The questionnaire was structured in five sections: demographic information, housing situation, residential area, nature (visit), and health. Mainly closed-ended questions were used to explore the above-mentioned objectives. For a detailed overview of the questionnaire, see Supplementary Data 1: **questionnaire**.

#### Demographic Information

Participants answered questions regarding age, gender, nationality, living situation (number of roommates), highest obtained educational degree (elementary school, secondary education, university of applied sciences bachelor's, university bachelor's, university master's, or post-university), work situation (working, student, retired, temporarily unemployed due to corona crisis, unemployed, job seeker, disabled, and sick), and remote working before and during C19CM (never, once a week, part of the time, always, and not applicable).

#### Housing Situation—Satisfaction and Characteristics

Questions were asked about the respondent's current housing situation. These were developed in cooperation with the department of housing from the Flemish government. Participants were asked about the type of housing they currently live in (open building, semi-detached building, closed building, apartment low-rise/high-rise, room, and studio), the belonging facilities (garden private/communal, courtyard, balcony, garage, or private parking), the size of the different rooms in the house (too small, small, medium, large, and too large), and their housing satisfaction before and during C19CM (very satisfied, satisfied, rather satisfied, rather dissatisfied, dissatisfied, and very dissatisfied).

#### Residential Area—Satisfaction and Infrastructure

In addition to questions about the housing situation, participants were asked to indicate their satisfaction about the residential area—before C19CM and at the present time. Questions were asked based on statements that were answered on a 6-point scale (1 = totally agree, 6 = not agree at all).

#### Nature—Frequency and Experience

Participants were asked about the presence of nature in their life. Nature was broadly defined: ranging from a green terrace/balcony or garden to nature in the environment such as a (city) park, nature reserve, forest, field, meadow, pond, river, sea, to beach. Respondents who went into nature were asked questions about the frequency (several times a day, once a day, several times a week, once or twice a week, and less than once a week) and motives (to hike/sport, the silence, social contact, boredom, etc.) before and during C19CM. Additionally, questions were presented about how important they find nature for their health (ranging from very important to not at all). Finally, participants were asked how they felt after their nature visit. Eleven statements were presented with seven answer options ranging from “totally agree” to “totally disagree.” Respondents who did not go into nature were introduced questions about their possible use of nature indoors (houseplants, green view, nature images/documentaries, nature sounds, and nature books).

#### Health

To obtain an indication of how respondents felt at the present time, questions were asked regarding their mental and physical health containing six respond categories (very healthy, healthy, rather healthy, rather not healthy, not healthy, and not at all healthy).

### Data Analysis

The data were analyzed using the software program SPSS. A number of steps were taken to obtain the results. To explore the data, a univariate analysis was carried out based on frequency tables. An overview of the variables used can be found in Table 1. Next, a multiple regression analysis was carried out. Based on an ordinal logistic regression, several independent variables (gender, age, educational attainment, mental and physical health, private garden, satisfaction with home and living environment, and sufficient green space in the living environment) were associated with the dependent variable “frequency of nature visit during C19CM.” In this way, we were able to detect to what extent the independent variables explain how often the participants visit nature. The results in Table 2 are presented using the Exp(B) coefficient or odds ratio, with a 95% confidence interval.

**Table 1:** Variables overview of frequencies (*N* = 11,352).

**Variable**	**Categories**	***N***	**%**	**Cum. %**
Frequency of nature visit (during COVID-19)	Not	550	4.8	4.8
	Less than once a week	166	1.5	6.3
	Once or twice a week	742	6.5	12.8
	Several times a week	2,442	21.5	34.4
	Once a day	3,534	31.1	65.5
	Multiple times a day	3,918	34.5	100
Gender	Man	3,568	31.4	31.4
	Woman	7,742	68.2	99.6
	Other	41	0.4	100
Age (in years)	12–18	81	0.7	0.7
	19–30	1,563	13.8	14.5
	31–40	2,356	20.8	35.2
	41–50	2,569	22.6	57.9
	51–65	3,642	32.1	89.9
	65+	1,141	10.1	100
Education level	Low	2,685	23.7	23.7
	High	8,562	75.4	99.1
	Other	105	0.9	100
Work situation (during COVID-19)	Working	7,361	64.8	64.8
	Not working	3,991	35.2	100
Housemates < 12 years	Yes	2,403	21.2	21.2
	No	8,949	78.8	100
Home satisfaction (during COVID-19)	Satisfied	10,678	94.1	94.1
	Not satisfied	674	5.9	100
Neighborhood satisfaction (during COVID-19)	Satisfied	10,556	93	93
	Not satisfied	796	7	100
Physical health	Healthy	10,263	90.4	90.4
	Unhealthy	1,050	9.2	99.7
	Missing	39	0.3	100
Mental health	Healthy	9,628	84.8	84.8
	Unhealthy	1,655	14.6	99.4
	Missing	69	0.6	100
Access to private garden	Yes	8,978	79.1	79.1
	No	2,374	20.9	100
Access to communal garden	Yes	446	3.9	3.9
	No	10,906	96.1	100
Sufficient green in neighborhood	Yes	8,965	79	79
	No	2,372	20.9	99.9
	Not applicable	15	0.1	100
Green and squares in neighborhood well-maintained	Yes	9,081	80	80
	No	131	12.3	92.2
	Not applicable	880	7.8	100

*COVID-19, coronavirus disease 2019*.

**Table 2:** Ordinal logistic regression “frequency of nature visit during C19CM” (*N* = 10,267; *p < 0.05, **p < 0.01, ***p < 0.001).

**Variable**	**Exp(B)**	**Lower (95% CI)**	**Upper (95% CI)**
(Ref = man)			
Woman	1.345***	1.246	1.452
(Ref = 65+ years old)			
12–18 years	0.652*	0.427	0.996
19–30 years	0.847*	0.722	0.993
31–40 years	1.010	0.858	1.190
41–50 years	0.999	0.856	1.166
51–65 years	1.055	0.918	1.211
(Ref = low education level)			
High education level	1.513***	1.386	1.651
(Ref = not working during C19CM)			
Working	0.884**	0.810	0.965
(Ref = housemates < 12 years old)			
No housemates < 12 years	0.776***	0.700	0.861
(Ref = dissatisfied with home during C19CM)			
Satisfied with home	1.366***	1.166	1.600
(Ref = dissatisfied with neighborhood during C19CM)			
Satisfied with neighborhood	1.133	0.974	1.319
(Ref = physically unhealthy)			
Physically healthy	1.458***	1.283	1.657
(Ref = mentally unhealthy)			
Mentally healthy	1.312***	1.180	1.458
(Ref = access to private garden)			
No access to private garden	0.454***	0.412	0.499
(Ref = access to communal garden)			
No access to communal garden	0.854	0.708	1.030
(Ref = insufficient green in neighborhood)			
Sufficient green in neighborhood	1.708***	1.556	1.874
(Ref = green and squares in neighborhood poorly maintained)			
Green and squares in neighborhood well-maintained	1.107	0.995	1.232
Coefficient of determination = pseudo *R*^2^(Nagelkerke)	10%		

*C19CM, coronavirus disease 2019 confinement measures*.

## Results

### Descriptive Analysis

Of the respondents, 95.1% went into nature during C19CM in Belgium. More than one third (36.3%) went several times a day. Of the respondents who visit nature during C19CM, 59.5% do so more often than before. Having more time due to the confinement measures (76.3%) appears to be the most important reason followed by an alternative to sitting inside (71.2%) and to exercise (68.5%). Contrary to this, the presence of too many people in nature was reported as the main reason (31.4%) for not going into nature, followed by being afraid of a possible contamination with the coronavirus (19.5%).

The most popularly reported places for nature visits were people's own garden or terrace (84.2%), followed by parks or forests (66.5%). Walking was the most practiced activity in nature (90%).

Remarkably, nature receives a higher value during C19CM. Hence, 51.6% of the respondents who go into nature during C19CM experience nature in a more positive way than before. An overview of the main reasons can be found in Table 3. Only 7% of all respondents experience nature more negatively than before C19CM. An overview of the main reasons to experience nature in a more negative way can be found in Table 4. A significant difference was found with respect to educational level (*X*^2^ = 49.695; *p* < 0.05). More positive feelings toward nature were reported among higher-educated respondents.

**Table 3:** Main reasons to experience nature in a more positive way than before C19CM (*N* = 5,856).

**Reason**	**% that named as reason**
Thankful for being able to go outside instead of constantly staying inside	88.2
Being able to go into nature for a longer period of time	42.4
Newly discovered stimuli or elements in nature	30.9
Grown connection with nature	30.7

*C19CM, coronavirus disease 2019 confinement measures*.

**Table 4:** Main reasons to experience nature in a more negative way than before C19CM (*N* = 759).

**Reason**	**% that named as reason**
Too crowded outside	67.8
It's no longer allowed to sit on a bench or in the grass	37.2
Fear of getting infected with COVID-19	34.9

*C19CM, coronavirus disease 2019 confinement measures*.

### Multiple Regression: Ordinal Logistic Regression

Since 95% of the respondents went into nature, the analysis investigated the variables related to the frequency of nature visits. An overview of these variables can be found in Table 2. Consequently, we identified three different themes associated with frequency of nature visit.

Firstly, frequency of nature visits is associated with the living environment. The analysis showed that respondents who are satisfied with their own home are more likely to go into nature several times a day than those who are dissatisfied with their home (*X*^2^ = 1,366; *p* < 0.05). However, satisfaction with one's home neighborhood does not appear to have a significant effect on the frequency of nature visits. Subsequently, respondents with a private garden are more likely to visit nature several times a day than respondents without a garden. This correlation is statistically significant according to the chi-square test (*X*^2^ = 584.154; *p* < 0.05). However, it should be noted that nature was broadly defined in this study, including own garden. Therefore, one may spend time in one's own garden when indicating visiting nature several times a day. Lastly, results showed that respondents who are satisfied with their residential area are more likely to visit nature several times a day (37.1%), compared with respondents who are dissatisfied (24%). This association was significant (*X*^2^ = 107,540; *p* < 0.05).

Secondly, we could identify three different demographic factors associated with frequency of nature visit: age, sex, and educational level. Regarding age, 7.4% of the respondents over the age of 65 do not go into nature during the C19CM. This is a significant lower percentage than among the younger age groups, of which a larger percentage does visit nature. However, people over the age of 65 who go into nature go more frequently than the age group 12–30 years. The other age categories do not appear to differ significantly from this reference group. Furthermore, women visit nature more often than men, though it must be acknowledged that our sample had an overrepresentation of women. Lastly, the analysis indicated that lower-educated people go into nature less frequently than higher-educated people (*X*^2^ = 133.316; *p* < 0.05). Of the lower-educated respondents, 91.1 went into nature during C19CM as compared with 96.5% of the higher-educated ones. Even after verification for age and sex, this association was still found to be significant [Exp(B) = 1,513; 95% CI = (1,386; 1,651)].

Lastly, subjective mental and physical health are associated with frequency of nature visits. The chi-square test indicates that respondents who feel mentally healthier are more likely to go into nature than those who indicate that they feel “not at all healthy”; 44.7% of the “very healthy” respondents go into nature several times a day, while only 23.1% of those who indicate that they feel “not at all healthy” do so (*X*^2^ = 207.405; *p* < 0.05). In terms of physical health and nature visits, we see a rather small but significant difference between physically healthy and non-healthy people (*X*^2^ = 71,687; *p* < 0.05). Of the respondents who feel physically healthy, 36.7% go into nature several times a day, compared with 31.9% of the people who do not feel healthy. However, our sample contained an overrepresentation of people indicating feeling healthy. An important difference was found considering health and level of education. Less-educated people reported feeling less physically healthy (88.5% compared with 91.5%) and slightly less mentally healthy (82.7% compared with 86.2%) than highly educated people.

## Discussion

We explored to which extent people visit nature (more often) during C19CM and which factors contribute to this. Half of the participants indicated going into nature more often than before C19CM. These findings complement research elsewhere in Europe (17, 39) and Asia (16).

This study can be embedded in the broader definition of nature contact from Frumkin (7). This study focused on one dimension of contact with nature, namely, visiting and going into nature. The results show a significant relationship between the frequency of nature visits and home environment, age, gender, socioeconomic status, and health.

Having more time due to the C19CM seems to stimulate respondents to visit nature more often than before (14, 30, 39). In line with previous research, gardens were reported as the most popular place, followed by parks (17). In addition, respondents with a private garden were more likely to visit nature several times a day.

More than half of the people experienced nature more positively during C19CM than before. Participants reported feeling less anxious, more relaxed, positive, fitter, and happier after visiting nature. The benefits of exposure to nature are well-highlighted in some previous work for reducing stress and anxiety (40) and improving physical and mental health (6, 41) and the general well-being (5, 42).

The majority of the respondents consider visiting nature important for their health, and this seems to have increased during C19CM. This is in accordance with the research from Lopez et al. (43). Nature helps to maintain social relationships during C19CM, as the younger respondents reported visiting nature more often than before to walk together with friends. It is known that spending time with others in nature can build social capital and also improve social cohesion (44). In this way, visiting nature may mitigate the negative effects of social isolation on mental health (45, 46), an effect that may have been especially important during lockdown (44, 47).

The level of high educated respondents in our sample was significantly higher than in the general population. Despite this response bias, the results show that less-educated people are less likely to go into nature during the C19CM and experience nature as less positive. In addition, we found a positive association between home satisfaction and nature visits that may also be explained by more favorable housing facilities for better-educated people. People with low levels of education are more likely to live in small dwellings (48) where the quality of access to nature may wane (49), and they report more often feeling unhealthy (50).

The present study is subject to several limitations. Firstly, a key limitation was that we did not detail enough in the questions which type of nature the respondents were visiting; hence, we could not clearly see whether this was, e.g., public (park, forest, field, etc.) or private nature (garden and green terrace/balcony). Perhaps, nature was defined too broadly. Due to the questioning and data analysis, we cannot specify how often people visit a particular place. This would have been an added value since previous research has shown how benefits can differ based on the type of nature (22). Secondly, our sample is not representative for the general population of Flanders. Therefore, results mainly apply within the characteristics of the sample. Generalization to a wider population remains speculative. There is an underrepresentation of low-educated and vulnerable groups who feel less healthy and a small underrepresentation of men. Future research should seek to achieve a more diverse sample. A mixed-method research design could be used to achieve this, in which qualitative research is necessary to reach more vulnerable groups. Furthermore, we must take the weather conditions into account. At the time of the survey, there was exceptionally good weather in Belgium, which may have possibly influenced the frequency of nature visits. Finally, we can question whether the behavior toward nature persists or whether this was only the case at the start of the confinement measures. A follow-up survey could verify this.

In sum, this study investigated the frequency of nature visits during the C19CM in Belgium and explored how people experienced nature. The results from this study are in accordance with previous studies who highlight the benefits of visiting nature for human health. People went into nature more often and reported positive feelings afterwards. The frequency of visiting nature was associated with several variables such as educational level, age, health, and living environment. Respondents with a higher educational attainment, who felt mentally and physically healthy, and were satisfied with their living environment went into nature more often.

This study highlights the importance of nearby green infrastructure. These findings show implications for policy makers to create more accessible green spaces and to keep these places accessible during C19CM, as it is considered a significant contribution to the general well-being and could serve as a coping strategy for emotion regulation.

## Data Availability Statement

The raw data supporting the conclusions of this article will be made available by the authors, without undue reservation.

## Author Contributions

AL: conceptualization and design of the study, coordination and supervision of the questionnaire distribution, data cleaning, analysis of results and interpretation, creation of figures and tables, and editing manuscript. SH: drafting and editing manuscript. AD: editing questionnaire, collaboration in survey distribution, data cleaning (conversion of online survey data into dataset), support with analysis, and editing manuscript. AS, LL, and RR: editing questionnaire, collaboration in survey distribution, and editing manuscript. HB: editing questionnaire and collaboration in survey distribution. HK: conceptualization and design of the study, coordination and supervision of the questionnaire distribution, data cleaning, analysis of results and interpretation, and editing manuscript. All authors contributed to the article and approved the submitted version.

## Conflict of Interest

The authors declare that the research was conducted in the absence of any commercial or financial relationships that could be construed as a potential conflict of interest.

## References

[1] Capaldi CA Dopko RL Zelenski JM. The relationship between nature connectedness and happiness: a meta-analysis. Front Psychol. (2014) 5:976. 10.3389/fpsyg.2014.00976 25249992 PMC4157607

[2] Cox DT Shanahan DF Hudson HL Fuller RA Anderson K Hancock S. Doses of nearby nature simultaneously associated with multiple health benefits. Int J Environ Res Public Health. (2017) 14:172. 10.3390/ijerph14020172 28208789 PMC5334726

[3] Romanelli C Cooper D Campbell-Lendrum D Maiero M Karesh WB Hunter D. Connecting Global Priorities: Biodiversity and Human Health: A State of Knowledge Review. Geneva; Montreal, QC: World Health Organistion/Secretariat of the UN Convention on Biological Diversity (2015).

[4] Richardson M Dobson J Abson DJ Lumber R Hunt A Young R. Applying the pathways to nature connectedness at a societal scale: a leverage points perspective. Ecosystems People. (2020) 16:387–401. 10.1080/26395916.2020.1844296

[5] Ives CD Abson DJ von Wehrden H Dorninger C Klaniecki K Fischer J. Reconnecting with nature for sustainability. Sustain Sci. (2018) 13:1389–97. 10.1007/s11625-018-0542-9 30220917 PMC6132401

[6] Vanaken GJ Danckaerts M. Impact of green space exposure on children's and adolescents' mental health: a systematic review. Int J Environ Res Public Health. (2018) 15:2668. 10.3390/ijerph15122668 30486416 PMC6313536

[7] Frumkin H Bratman GN Breslow SJ Cochran B Kahn PH Jr Lawler JJ. Nature contact and human health: a research agenda. Environ Health Perspect. (2017) 125:075001. 10.1289/EHP1663 28796634 PMC5744722

[8] Lauwers L Bastiaens H Remmen R Keune H. Nature's contributions to human health: a missing link to primary health care? A scoping review of international overview reports and scientific evidence. Front Public Health. (2020) 8:52. 10.3389/fpubh.2020.00052 32257986 PMC7093563

[9] Shanahan DF Bush R Gaston KJ Lin BB Dean J Barber E. Health benefits from nature experiences depend on dose. Sci Rep. (2016) 6:28551. 10.1038/srep28551 27334040 PMC4917833

[10] Vanhove MPM Thys S Decaestecker E Antoine-Moussiaux N De Man J Hugé J. Global change increases zoonotic risk, COVID-19 changes risk perceptions: a plea for urban nature connectedness. Cities Health. (2020) 4. 10.1080/23748834.2020.1805282

[11] Austenfeld JL Stanton AL. Coping through emotional approach: a new look at emotion, coping, health-related outcomes. J Pers. (2004) 72:1335–64. 10.1111/j.1467-6494.2004.00299.x 15509285

[12] Berto R. The role of nature in coping with psycho-physiological stress: a literature review on restorativeness. Behav Sci. (2014) 4:394–409. 10.3390/bs4040394 25431444 PMC4287696

[13] Richardson M. Beyond restoration: considering emotion regulation in natural well-being. Ecopsychology. (2019) 11:123–9. 10.1089/eco.2019.0012

[14] Constandt B Thibaut E De Bosscher V Scheerder J Ricour M Willem A. Exercising in times of lockdown: an analysis of the impact of COVID-19 on levels and patterns of exercise among adults in Belgium. Int J Environ Res Public Health. (2020) 17:4144. 10.3390/ijerph17114144 32532013 PMC7312512

[15] Gray S Kellas A. Covid-19 has highlighted the inadequate, and unequal, access to high quality green spaces (2020). Retrieved from: https://blogs.bmj.com/bmj/2020/07/03/covid-19-has-highlighted-the-inadequate-and-unequal-access-to-high-quality-green-spaces/

[16] Lu Y Zhao J Wu X Lo SM. Escaping to nature in pandemic: a natural experiment of COVID-19 in Asian cities (2020) 1−35. 10.31235/osf.io/rq8sn

[17] Ugolini F Massetti L Calaza-Martínez P Cariñanos P Dobbs C Ostoic SK. Effects of the COVID-19 pandemic on the use and perceptions of urban green space: an international exploratory study. Urban Forestry Urban Greening. (2020). 10.1016/j.ufug.2020.126888 33100944 PMC7566824

[18] Frumkin H Fox J. Contact with nature. In: Making Healthy Places. Washington, DC: Island Press (2011). p. 229–43.

[19] Keniger LE Gaston KJ Irvine KN Fuller RA. What are the benefits of interacting with nature? Int J Environ Res Public Health. (2013) 10:913–35. 10.3390/ijerph10030913 23466828 PMC3709294

[20] Friedman B Freier NG Kahn PH Jr Lin P Sodeman R. Office window of the future?—Field-based analyses of a new use of a large display. Int J Hum Comput Stud. (2008) 66:452–65. 10.1016/j.ijhcs.2007.12.005

[21] Beute F Andreucci MB Lammel A Davies Z Glanville J Keune H. Types and characteristics of urban and peri-urban green spaces having an impact on human mental health and wellbeing. EKLIPSE report (2020). Retrieved from: https://www.eklipse-mechanism.eu/

[22] WHO Regional Office for Europe. Urban Green Spaces and Health. Copenhagen: WHO Regional Office for Europe (2016).

[23] Huang C Wang Y Li X Ren L Zhao J Hu Y. Clinical features of patients infected with 2019 novel coronavirus in Wuhan, China. Lancet. (2020) 395:497–506. 10.1016/S0140-6736(20)30183-5 31986264 PMC7159299

[24] Sciensano. Coronavirus (2020). Available online at: https://www.sciensano.be/nl/gezondheidsonderwerpen/coronavirus#:~:text=Het%20coronavirus%20SARS%2DCoV%2D2,de%20oorspronkelijke%20naam%202019%2DnCoV (accessed December 21, 2020).

[25] World Health Organisation. WHO Director-General's opening remarks at the media briefing on COVID-19 (2020). Available online at: https://www.who.int/dg/speeches/detail/who-director-general-s-opening-remarks-at-the-media-briefing-on-covid-19-−11-march-2020 (accessed December 21, 2020).

[26] WHO. WHO coronavirus disease dashboard (2020). Available online at: https://covid19.who.int/ (accessed December 21, 2020).

[27] Anderson RM Heesterbeek H Klinkenberg D Hollingsworth TD. How will country-based mitigation measures influence the course of the COVID-19 epidemic? Lancet. (2020) 395:931–4. 10.1016/S0140-6736(20)30567-5 32164834 PMC7158572

[28] Lau H Khosrawipour V Kocbach P Mikolajczyk A Schubert J Bania J. The positive impact of lockdown in Wuhan on containing the COVID-19 outbreak in China. J Travel Med. (2020) 27:taaa037. 10.1093/jtm/taaa037 32181488 PMC7184469

[29] Nussbaumer-Streit B Mayr V Dobrescu AI Chapman A Persad E Klerings I. Quarantine alone or in combination with other public health measures to control COVID-19: a rapid review. Cochrane Database Systematic Rev. (2020) 8:CD013574. 10.1002/14651858.CD013574 33959956 PMC8133397

[30] Randler C Tryjanowski P Jokimäki J Kaisanlahti-Jokimäki ML Staller N. SARS-CoV2 (COVID-19) pandemic lockdown influences nature-based recreational activity: the case of birders. Int J Environ Res Public Health. (2020) 17:7310. 10.3390/ijerph17197310 33036351 PMC7579058

[31] Pfefferbaum B North CS. Mental health and the Covid-19 pandemic. N Engl J Med. (2020) 510–2. 10.1056/NEJMp2008017 32283003

[32] Rajkumar RP. COVID-19 and mental health: a review of the existing literature. Asian J Psychiatry. (2020) 52:102066. 10.1016/j.ajp.2020.102066 32302935 PMC7151415

[33] Ammar A Chtourou H Boukhris O Trabelsi K Masmoudi L Brach M. COVID-19 home confinement negatively impacts social participation and life satisfaction: a worldwide multicenter study. Int J Environ Res Public Health. (2020) 17:6237. 10.3390/ijerph17176237 32867287 PMC7503681

[34] Ammar A Brach M Trabelsi K Chtourou H Boukhris O Masmoudi L. Effects of COVID-19 home confinement on eating behaviour and physical activity: results of the ECLB-COVID19 international online survey. Nutrients. (2020) 12:1583. 10.3390/nu12061583 32481594 PMC7352706

[35] De Backer C Teunissen L Cuykx I Decorte P Pabian S Gerritsen S. An evaluation of the COVID-19 pandemic and perceived social distancing policies in relation to planning, selecting, and preparing healthy meals: an observational study in 38 countries worldwide. Front Nutrition. (2020) 7:621726. 10.3389/fnut.2020.621726 33614693 PMC7890074

[36] BELGIUM. Coronavirus: versterkte maatregelen (2020). Retrieved from: https://www.belgium.be/nl/nieuws/2020/coronavirus_versterkte_maatregelen

[37] Bratman GN Anderson CB Berman MG Cochran B De Vries S Flanders J. Nature and mental health: An ecosystem service perspective. Sci Advances. (2019) 5:eaax0903. 10.1126/sciadv.aax0903 31355340 PMC6656547

[38] Bryman A. Social Research Methods. New York, NY: Oxford University Press (2012).

[39] Venter Z Barton D Gundersen V Figari H Nowell M. Urban nature in a time of crisis: recreational use of green space increases during the COVID-19 outbreak in Oslo, Norway. Environ Res Letters. (2020). 10.1088/1748-9326/abb

[40] Nutsford D Pearson AL Kingham S. An ecological study investigating the association between access to urban green space and mental health. Public Health. (2013) 127:1005–11. 10.1016/j.puhe.2013.08.016 24262442

[41] Fong KC Hart JE James P. A review of epidemiologic studies on greenness and health: updated literature through 2017. Curr Environ Health Rep. (2018) 5:77–87. 10.1007/s40572-018-0179-y 29392643 PMC5878143

[42] Hartig T Mitchell R De Vries S Frumkin H. Nature and health. Annu Rev Public Health. (2014) 35:207–28. 24387090 10.1146/annurev-publhealth-032013-182443

[43] Lopez B Kennedy C McPhearson T. Parks are Critical Urban Infrastructure: Perception and Use of Urban Green Spaces in NYC During COVID*-*19. (2020) 2020:2020080620. 10.20944/preprints202008.0620.v2

[44] Samuelsson K Barthel S Colding J Macassa G Giusti M. Urban nature as a source of resilience during social distancing amidst the coronavirus pandemic. (2020). 10.31219/osf.io/3wx5a

[45] Cartwright BD White MP Clitherow TJ. Nearby nature ‘buffers’ the effect of low social connectedness on adult subjective wellbeing over the last 7 days. Int J Environ Res Public Health. (2018) 15:1238. 10.3390/ijerph15061238 29895738 PMC6025411

[46] Yang Y Wang L Passmore HA Zhang J Zhu L Cai H. Viewing nature scenes reduces the pain of social ostracism. J Soc Psychol. (2020) 161:197–215. 10.1080/00224545.2020.1784826 32633650

[47] Singh S Roy MD Sinha C Parveen S Sharma G Joshi G. Impact of COVID-19 and lockdown on mental health of children and adolescents: A narrative review with recommendations. Psychiatry Res. (2020) 293:113429. 10.1016/j.psychres.2020.113429 32882598 PMC7444649

[48] Singu S Acharya A Challagundla K Byrareddy SN. Impact of social determinants of health on the emerging COVID-19 pandemic in the United States. Front Public Health. (2020) 8:406. 10.3389/fpubh.2020.00406 32793544 PMC7385373

[49] Elliott JR Korver-Glenn E Bolger D. The successive nature of city parks: making and remaking unequal access over time. City Community. (2019) 18:109–27. 10.1111/cico.12366

[50] Marmot M Allen J Bell R Bloomer E Goldblatt P. WHO European review of social determinants of health and the health divide. Lancet. (2012) 380:1011–29. 10.1016/S0140-6736(12)61228-8 22964159

