# Supplementary material for: Vitamin Nature: How Coronavirus Disease 2019 Has Highlighted Factors Contributing to the Frequency of Nature Visits in Flanders, Belgium
Source: Front Public Health. 2021 May 11;9:646568. doi: 10.3389/fpubh.2021.646568 (PMC8144317; doi:10.3389/fpubh.2021.646568)
Supplement: Supplementary file 1 [file Table_1.DOCX]

Supplementary Material

# Supplementary Data 1: questionnaire

**A_General questions**

**A_1 What is your age**

... year (Restriction: between 0 and 110 years)

**A_2 Which gender do you identify yourself with?**

- Man
- Woman
- Other

**A_3 Where do you live (zip code)?**

... (Restriction: exactly 4 digits)

**A_4 Which nationality do you have?**

- Belgian
- Non-Belgian, namely (complete): .........

**A_5 What nationality did your mother have when she was born?**

- Belgian
- Non-Belgian, namely (complete): .........

**A_6 What nationality was your father when he was born?**

- Belgian
- Non-Belgian, namely (complete): .........

**A_7 Do you live alone or do you have roommates?**

- I live alone go to question A_9
- I have roommates

**A_8 How many roommates do you have (excluding yourself)?**

- Adults over 70 years old :................... (Restriction:3 digits)
- Adults between 18 and 70 years old :............... (Restriction:3 digits)
- Children between 12 and under 18 years old:....... (Restriction:2 digits)
- Children under 12 years of age:......................... (Restriction:2 digits)

**A_9 What is the highest level of education you have obtained? (If you are still a student, please indicate the degree you are studying for)**

- Primary education
- Secondary education: ASO/KSO
- Secondary education: BSO
- Secondary education: TSO
- University of Applied Sciences: bachelor
- University: bachelor/master (candidate/licentiate)
- Post-university: doctor
- Other

**A_10 Are you currently working, during the corona crisis (at work or from home)?**

- Yes => go to question A_12
- No

**A_11 I am:**

- Student
- Retirement
- Temporarily unemployed due to the corona crisis
- Unemployed / job seeker
- Disabled
- Sick
- Other: …..

**=> go to question B_1**

**A_12 How often did you normally work from home before the corona crisis?**

- Never
- 1 day a week
- Part of my working time
- Always
- Not Applicable

**A_13 How often do you work from home during the corona crisis?**

- Never
- 1 day a week
- Part of my working time
- Always
- Not Applicable

**B_House**

**The following questions are about your current living situation. This is the house you are currently living in.**

**B_1 What type of home do you live in?**

- Single-family dwelling (house) - open buildings
- Single-family dwelling (house) - semi-detached buildings
- Single-family house (row house) - closed buildings
- Apartment in low-rise (in building with a maximum of 4 floors)
- Apartment in high-rise (in building with more than 4 floors)
- Room
- Studio
- Other (complete):...

**B_2 The house where I am currently staying has a ...... (multiple answers are possible)**

- Private garden
- Communal garden (e.g. for the residents of the apartment building)
- Course
- Balcony
- Garage or private parking
- None of the above

**B_3 This questions the size of your house and the different rooms. Indicate what is applicable for you.**

|  | Too Small | Small | Medium | Large | Too Large | Not applicable |
| --- | --- | --- | --- | --- | --- | --- |
| My home is … |  |  |  |  |  |  |
| My dining area is … |  |  |  |  |  |  |
| My kitchen is … |  |  |  |  |  |  |
| My storage room is … |  |  |  |  |  |  |
| My bedroom is … |  |  |  |  |  |  |
| My bathroom is … |  |  |  |  |  |  |
| My laundry is … |  |  |  |  |  |  |
| My office is … |  |  |  |  |  |  |

**B_4 How satisfied were you with your home before the corona crisis?**

- Very Satisfied
- Satisfied
- Rather satisfied
- Rather dissatisfied
- Dissatisfied
- Very dissatisfied

**B_5 How satisfied are you with your home at the moment, during the corona crisis?**

- Very Satisfied
- Satisfied
- Rather satisfied
- Rather dissatisfied
- Dissatisfied
- Very dissatisfied

**C_Neighbourhood**

**The following questions are about the neighbourhood in which you currently live.**

**C_1 To what extent do you agree with the following statements about your neighbourhood? These statements relate to the period before the corona crisis. Indicate.**

|  | Completely agree | Agree | Rather agree | Rather disagree | Disagree | Completely disagree | Not applicable |
| --- | --- | --- | --- | --- | --- | --- | --- |
| I live in a nice neighbourhood |  |  |  |  |  |  |  |
| There is enough space between the buildings |  |  |  |  |  |  |  |
| Children can play safely in the neigbourhood |  |  |  |  |  |  |  |
| There is plenty of greenery nearby |  |  |  |  |  |  |  |
| There is too much green in the neighbourhood |  |  |  |  |  |  |  |
| There are plenty of squares and parks nearby |  |  |  |  |  |  |  |
| The greenery and squares nearby are well maintained |  |  |  |  |  |  |  |
| The streets and footpaths in my neighbourhood are in good condition |  |  |  |  |  |  |  |
| There are plenty of footpaths nearby |  |  |  |  |  |  |  |
| There are plenty of cycle paths nearby |  |  |  |  |  |  |  |
| There is not too much traffic nearby |  |  |  |  |  |  |  |
| There is no speeding in the neighbourhood |  |  |  |  |  |  |  |
| There is plenty of parking space nearby |  |  |  |  |  |  |  |
| When it is dark, the neighbourhood is sufficiently illuminated |  |  |  |  |  |  |  |
| I live close enough to family, friends and acquaintances |  |  |  |  |  |  |  |
| I have sufficient contact with my neighbourhood |  |  |  |  |  |  |  |
| There are plenty of meeting places in my neighbourhood |  |  |  |  |  |  |  |
| My house is well located, sufficiently accessible |  |  |  |  |  |  |  |
| I live near my work |  |  |  |  |  |  |  |
| I live near a school |  |  |  |  |  |  |  |
| I live near stores |  |  |  |  |  |  |  |

**C_2 How satisfied were you with the neighbourhood you live in, before the corona crisis?**

- Very Satisfied
- Satisfied
- Rather satisfied
- Rather dissatisfied
- Dissatisfied
- Very dissatisfied

**C_3 How satisfied are you at the moment with the neighbourhood you live in, during the corona crisis?**

- Very Satisfied
- Satisfied
- Rather satisfied
- Rather dissatisfied
- Dissatisfied
- Very dissatisfied

**D_ Nature**

**The following questions deal with the presence of nature during the corona crisis.**

**D_1 Do you ever go into nature* at the moment, during the coronacrisis?**

**Nature can be interpreted broadly, ranging from a green terrace/balcony or garden to nature in your environment such as a (city) park, nature reserve, forest, field, meadow, pond, river, sea, beach ...*

- Yes -> go to question D_6
- No -> go to question D_2

**D_2 Why don't you go into nature during the coronacrisis? Indicate, multiple answers are possible.**

|  | Indicate |
| --- | --- |
| I never go into nature |  |
| Because I may not or cannot go into nature through corona (for example: I am in quarantine for my health and/or that of others). |  |
| Because I don't dare to go into nature (because I'm afraid of getting infected or possibly infecting others). |  |
| Because I no longer pass nature (on foot, by bike) on my way to work, school or leisure activities like before the corona crisis. |  |
| Because some nature spots in my habitat are closed off by the coronavirus. |  |
| Because there are too many people outside (too busy). |  |
| Because at the moment I don't have time to go into nature. |  |
| Because I don't have a partner/friend who goes with me into nature. |  |
| Other (complete):... |  |

**D_3 Are you looking for other ways to enjoy nature indoors? For example, do you have houseplants, do you look at images of nature (on TV, in a book) or do you enjoy a green view?**

- Yes -> go to D_4
- No -> go to D_18

**D_4 How do you deal with nature indoors? Several answers are possible.**

- I have indoor plants
- I enjoy a green view (plants and/or animals outside)
- I watch nature images and/or nature documentaries on TV (or cell phone, tablet...).
- I listen to nature sounds
- I read nature books
- Other (complete): ...

**D_5 Are you now more occupied with nature indoors than before the corona crisis?**

- Yes, I am,
- No
- As many as before

🡪 **Go to D_18**

**D_6 If you go into nature, where do you go? Indicate, multiple answers are possible.**

|  | Indicate |
| --- | --- |
| Private garden/terrace with (view of) greenery |  |
| Green living environment (streets, avenues, avenues) |  |
| City park/public garden |  |
| Nature reserve or forest |  |
| Fields and meadows |  |
| Pools and ponds (small water elements) |  |
| Lakes and rivers (large water elements) |  |
| Sea, beach |  |
| Other (complete):... |  |

**D_7 Why are you going into nature? Indicate your reasons for the period before and during the coronacrisis.**

|  | Reasons before coronacrisis | Reasons during coronacrisis |
| --- | --- | --- |
| To Hike |  |  |
| To do Sports |  |  |
| For the peace and quiet |  |  |
| To be alone |  |  |
| For social contact |  |  |
| For the healthy environment |  |  |
| To enjoy nice weather |  |  |
| For gardening |  |  |
| To study nature, observe |  |  |
| For experiencing nature, experiencing natural stimuli |  |  |
| To take a break while working |  |  |
| To do my job |  |  |
| Out of boredom, as a pastime |  |  |
| Other (describe reason):...... |  |  |

**D_8 How often do you go into nature during the coronacrisis?**

- Several times a day
- Once a day
- Several times a week
- Once or twice a week
- Less than once a week

**D_9 Do you go into nature more often now than before the coronacrisis?**

- Yes, I go into nature more often now than before the coronacrisis -> go to D_10
- No, I go now less often than before the coronacrisis into nature -> go to D_11
- I now go into nature as often as before the coronacrisis -> go to D_12

**D_10 Why do you go into nature more often now than before the coronacrisis? Indicate, multiple answers are possible.**

|  | Indicate |
| --- | --- |
| Because I have more time now |  |
| Because it is recommended by the government agencies |  |
| Because this way I can still get in touch with  others (social contact) |  |
| Because it's good for my  resistance/health, and this is important  during the corona crisis |  |
| Because I can still go outside and don't have to stay inside |  |
| Because I can move outside |  |
| Other (complete): ... |  |

**D_11 Why do you go into nature less often than before the coronacrisis? Indicate, several answers are possible.**

|  | Indicate |
| --- | --- |
| Because I no longer pass nature (on foot, by bike) on my way to work, school or leisure like before the corona crisis. |  |
| Because some nature spots in my habitat are closed off by the coronavirus. |  |
| Because there are too many people outside (too crowded) |  |
| Because at the moment I have less time to go into nature |  |
| Because I don't have a partner/boyfriend who goes with me into nature. |  |
| Because this way I have less chance of being infected by others with the coronavirus |  |
| Other (complete):… |  |

**D_12 To what extent do you experience nature in your living environment differently now than before the coronacrisis?**

- Much more negative (reference to D_13)
- More negative (reference to D_13)
- Rather more negative (reference to D_13)
- The same (reference to question D_15)
- Rather positive (reference to D_14)
- More positive (reference to D_14)
- Much more positive (reference to D_14)

**D_13 Why do you experience nature more negatively now than before the coronacrisis? Indicate, several answers are possible.**

|  | Indicate |
| --- | --- |
| Because I'm afraid of being infected by others outside with the coronavirus. |  |
| Because I am more vulnerable to the effects of the coronavirus due to another disease. |  |
| Because I myself show corona-like symptoms and am afraid to infect others. |  |
| Because it is too crowded outside |  |
| Because I am no longer allowed to sit on a bench or in the grass |  |
| Because the presence of the police bothers or deters me |  |
| Because I don't know what is or isn't allowed |  |
| Because the coronavirus comes from nature |  |
| Other (complete): ... |  |

**D_14 Why do you experience nature more positively now than before the coronacrisis? Indicate, several answers are possible.**

|  | Indicate |
| --- | --- |
| Because I can now go into nature for a longer period of time |  |
| Because now I feel more connected with nature |  |
| Because I'm grateful that I can go outside and therefore don't have to stay inside. |  |
| Because I am grateful that I can move outside |  |
| Because I've discovered new things in nature (visuals, smells, sounds) |  |
| Because I'm glad I can still get in touch with others in this way. |  |
| Other (complete): ... |  |

**D_15 How important did you think it was for your health to go into nature before the coronacrisis?**

- Not important at all
- Not important
- Rather not important
- Rather important
- Important
- Very important

**D_16 How important do you think it is for your health to go into nature during the coronacrisis?**

- Not important at all
- Not important
- Rather not important
- Rather important
- Important
- Very important

**D_17 How do you feel after being outside in nature, during the corona crisis? Indicate to what extent you agree with the following statements.**

|  | Completely agree | Agree | Rather agree | Rather Disagree | Disagree | Completely disagree | I don't know |
| --- | --- | --- | --- | --- | --- | --- | --- |
| I feel fitter than before I went into nature |  |  |  |  |  |  |  |
| I am more positive than before I went into nature |  |  |  |  |  |  |  |
| I feel relaxed and feel less stress than before I went into nature |  |  |  |  |  |  |  |
| I have more energy than before I went into nature |  |  |  |  |  |  |  |
| I feel happier than before I went into nature |  |  |  |  |  |  |  |
| I can concentrate better than before I went into nature |  |  |  |  |  |  |  |
| I feel more vulnerable than before I went into nature |  |  |  |  |  |  |  |
| I feel more anxious than before I went into nature |  |  |  |  |  |  |  |
| I have more stress than before I went into nature |  |  |  |  |  |  |  |
| I feel more insecure than before I went into nature |  |  |  |  |  |  |  |
| I feel the same as before |  |  |  |  |  |  |  |

**E_ Health**

**The following questions are about your health.**

**E_1 How healthy do you feel physically at the moment?**

- Very healthy
- Healthy
- Rather healthy
- Rather not healthy
- Not healthy
- Not at all healthy

**E_2 How healthy do you feel in your head right now (mentally):**

- Very healthy
- Healthy
- Rather healthy
- Rather not healthy
- Not healthy
- Not at all healthy
